# A capillary blood ammonia bedside test following glutamine load to improve the diagnosis of hepatic encephalopathy in cirrhosis

**DOI:** 10.1186/1471-230X-11-134

**Published:** 2011-12-08

**Authors:** Saskia Ditisheim, Emiliano Giostra, Pierre R Burkhard, Nicolas Goossens, Gilles Mentha, Antoine Hadengue, Laurent Spahr

**Affiliations:** 1 Gastroenterology and Hepatology, University Hospitals and Faculty of Medicine, 4, Rue Gabrielle Perret-Gentil, 1211 Geneva, Switzerland; 2 Neurology, University Hospitals and Faculty of Medicine, 4, Rue Gabrielle Perret-Gentil, 1211 Geneva, Switzerland; 3 Transplantation Unit and Visceral Surgery, University Hospitals and Faculty of Medicine, 4, Rue Gabrielle Perret-Gentil, 1211 Geneva, Switzerland

## Abstract

**Background:**

Hepatic encephalopathy (HE) is a frequent and severe complication of cirrhosis. A single determination of ammonia in venous blood correlates poorly with neurological symptoms. Thus, a better biological marker is needed.

**Aim:**

To make a diagnosis of HE, we explored the value of ammonia in capillary blood, an equivalent to arterial blood, measured at bedside following an oral glutamine challenge.

**Methods:**

We included 57 patients (age 56 yrs; M/F: 37/20) with cirrhosis (alcoholic = 42; MELD score 13.8 [7-29], esophageal varices = 38) and previous episodes of HE (n = 19), but without neurological deficits at time of examination, and 13 healthy controls (age 54 yrs). After psychometric tests and capillary (ear lobe) blood ammonia measurements, 20 gr of glutamine was administered orally. Tests were repeated at 60 minutes (+ blood ammonia at 30'). Minimal HE was diagnosed if values were > 1.5 SD in at least 2 psychometric tests. Follow-up lasted 12 months.

**Results:**

The test was well tolerated (nausea = 1; dizziness = 1). Patients showed higher values of capillary blood ammonia over time as compared to controls (0'-30'-60 minutes: 75, 117, 169 versus 52, 59, 78 umol/L, p < 0.05). At baseline, 25 patients (44%) had minimal HE, while 38 patients (67%) met the criteria for HE at 60 minutes (chi^2^: p < 0.01). For the diagnosis of minimal HE, using the ROC curve analysis, baseline capillary blood ammonia showed an AUC of 0.541 (CI: 0.38-0.7, p = 0.6), while at 60 minutes the AUC was 0.727 (CI: 0.58-0.87, p < 0.006). During follow-up, 18 patients (31%) developed clinical episodes of HE. At multivariate analysis, the MELD score (1.12 [1.018-1.236]), previous episodes of HE (3.2[1.069-9.58]), but not capillary blood ammonia, were independent predictors of event.

**Conclusions:**

In patients with cirrhosis and normal neurological examination, bedside determination of ammonia in capillary blood following oral glutamine load is well tolerated and achieves a better diagnostic performance for minimal HE than basal capillary ammonia levels. However, capillary blood ammonia is a poor predictor of development of clinically overt HE.

## Background

Hepatic encephalopathy (HE) is a common complication of cirrhosis that affects quality of life, increases the risk of accidents, and is an independent predictor of poor outcome [1,2]. When neurological deficits are subtle but the neurological clinical examination is normal, a condition referred to as minimal HE [3], patients are exposed to a risk of developing clinical episodes of HE over time [4]. The presence of HE in cirrhosis is a prognostic marker of severity and a valid indication for liver transplantation, although it is not considered in the model for end-stage liver disease (MELD) score on which organ distribution is based in most liver transplant centres [5].

Neurological alterations observed in HE are postulated to result from the exposure of the brain to abnormally elevated concentrations of ammonia present in the general circulation in response to liver insufficiency and portosystemic collaterals [6]. Accordingly, high ammonia levels have been associated with large portosystemic collaterals such as esophageal varices in patients with cirrhosis [7]. However, ammonia determination is not currently accepted as a reliable marker to identify patients with HE [8]. Hyperammonemia arises from the production by colonic bacteria and the small intestine through an increased intestinal glutaminase activity [9]. Although the pathogenesis of HE is still incompletely elucidated, the ammonia hypothesis remain central [10] and a large number of experimental data support the role of hyperammonemia in the direct and indirect alterations of brain function that characterize HE [11].

Making a diagnosis of HE may be straightforward when a patient with cirrhosis presents with obvious neurological deficits such as altered consciousness, but it is much more challenging in the presence of more subtle neuropsychological or personality changes that are not uncommon in an outpatient population of cirrhotics (up to 62% in a recent report [12]). In fact, it is recommended to search for minimal HE in patients who complain of cognitive alterations, a disturbed sleep [13], or are exposed to an accident risk while driving or at their work-place. As neurological deficits associated with minimal HE are clinically subtle, this complication may be underdiagnosed and may negatively impact patients' management. Accordingly, minimal HE can be accessible to medical therapy that may improve quality of life and prevent the development of clinical episodes of overt HE.

In clinical practice, the available tools for the diagnosis of HE include clinical scales to assess the mental status, such as the West-Haven scale [14], and a number of psychometric tests to assess the presence of congnitive deficits [15]. Neuroradiological imaging is mostly directed at excluding other neurological disorders. Blood ammonia concentration in the context of HE is difficult to interpret, as the correlation between neurological symptoms and ammonia blood levels is variable, with a wide overlap across different stages of HE [8]. Some [16,17], but not all [7,18] studies report a closer correlation with arterial as compared to venous ammonia blood levels. The diagnosis of minimal HE is therefore challenging for the clinician who has to choose between a suboptimal biological test or a number of neuropsychological tests that may be time consuming, need to be adjusted for several parameters, and are subject to learning biases [3].

To improve the performance of blood ammonia for the diagnosis of minimal HE, we hypothesized that measuring ammonia in arterialized blood following oral glutamine-induced hyperammonemia and consecutive cognitive deterioration would increase the diagnostic yield of this biological test. Therefore, we explored the value of capillary blood ammonia measured at bedside following an oral glutamine challenge to unmask HE in patients with cirrhosis.

## Methods

### Patients

Between January 2008 and February 2009, a total of 60 patients with cirrhosis were considered eligible for this study. Thirty eight were outpatient, and 22 patients were hospitalized for investigation of a chronic liver disease or as part of an assessment for liver transplantation. The diagnosis of cirrhosis was based on histology in 54 patients, and based on clinical, biological and radiological alterations in the other 6 patients. Inclusion criteria were as follows: cirrhosis in a stable condition with mild to severe liver failure (MELD 10 to 30), age 18 to 70 yrs, with a grade 0 of HE according to West-Haven criteria [14], and a written informed consent to participate. Exclusion criteria were the following: clinically overt HE (grade I or higher), diagnosis of a neurological or psychiatric disease other than HE, current use of psychotropic medications including benzodiazepines, recent (< 3 months) modification in the routine treatment of HE including nonabsorbable disaccharides and antibiotics, active alcoholism, active or recent use of nonsteroidal antiinflammatory drugs that may affect intestinal mucosal barrier, diagnosis of severe gastroparesis, history of small bowel surgical resection, inability to perform psychometric tests, and absence of informed consent.

Three patients with grade 1 HE according to West-Haven criteria [14] on the day of the test were excluded. Table 1 provides the characteristics of the 57 enrolled patients. The majority were male with alcoholic cirrhosis with clinically significant portal hypertension invasively measured in 51 patients. Most patients had moderate to severe liver failure, and large portosystemic collateral veins were visible at imaging studies in 39 out of the 57 patients. Four patients had a transjugular intrahepatic shunt previously placed for refractory ascites (n = 2) or recurrent bleeding (n = 2). Chronic administration of nonabsorbable disaccharides or antibiotics were reported in 20 and 17 patients, respectively.

**Table 1:** Patients characteristics

Age (yrs)	56.4 [42-69]
Gender (M/F)	37/20
Etiology of cirrhosis: Alcoholic	42
Other	15
MELD score	13.8 [7-29]
Child class (A/B/C)	10/33/14
HVPG (mmHg)	16 [13-22]
Esophageal varices	38/57
Ascites	29/57
TIPS	4/57
Previous episodes of HE	19/57
Nonabsorbable disaccharides	20/57
Antibiotics	17/57

Abbreviations: MELD: model for end-stage liver disease; HVPG: hepatic venous pressure gradient. TIPS: transjugular intrahepatic portosystemic shunt.

### Design of the study

**Figure 1:**
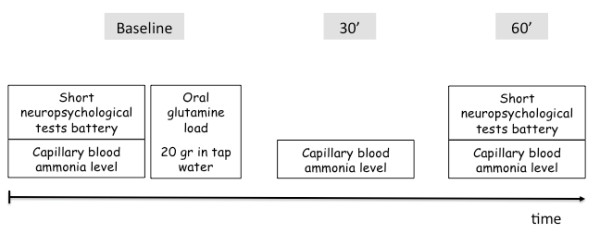
**Design of the study**.

Included patients were invited to undergo a baseline capillarized blood ammonia measurement prior to the performance of psychometric tests, followed by an oral glutamine load. Blood ammonia levels were again determined at 30 and 60 minutes, together with psychometric tests. Basing ourselves on peak ammonia levels in a previous study [19], we decided not to extend the observation period beyond 60 minutes. The study design is summarized in Figure 1.

### Psychometric tests

The diagnosis of minimal HE was based on psychometric tests that evaluate attention, executive function, speed of information processing and motor functions, as recommended in previously published guidelines [3]. To minimize the complexity of a comprehensive but demanding neuropsychiatric evaluation, we constructed a short battery of tests that included the Trail Making Test part A and B, as well as the Purdue Pegboard. Trail tests detect impairment in psychomotor speed and mental flexibility, both domains that are often impaired in HE [3]. Results are expressed in seconds. The Purdue Pegboard (Lafayette Instrument, IN, USA) is a validated method for evaluating the velocity and accuracy of fine movements of both upper extremities by measuring how quickly a subject can properly insert pins into grooved holes during a 30-second period [20]. Results are expressed in units (number of pins inserted during a given time period). The short psychometric tests battery was administered by trained hepatologists familiar with neuropsychological evaluation (SD, LS), under the supervision of a senior neurologist (PRB). The tests were performed while comfortably seated in a quiet and well-lit room, after a thorough explanation of the test and a practice run have been given to all patients. A modified version of the Trail Making Test A and B was used for the repeated evaluation at 60 minutes to avoid learning biases. All results were adjusted for age, gender and level of education using normative data. The presence of minimal HE was defined by the presence of at least 2 abnormal psychometric tests values (> 1.5 standard deviation), including the Trailmaking test B, in the absence of overt neurological manifestations, as reported [21]. Healthy subjects were also asked to perform Trail Making Test A and B at baseline and at 60 minutes.

### Oral glutamine challenge

In a fasting and metabolically stable state, patients were asked to ingest a solution of 20 gr of glutamine (Glutamin OX5, Fresenius-Kabi, Stans, Switzerland) dissolved in 50 ml tap water. Patients under chronic disaccharides or antibiotics remained so until the test. We chose this glutamine dosage as it induces a significant rise in blood ammonia following oral intake [22]. Ammonia was measured at bedside using a point-of-care testing (POCT) device (PocketChem Ammonia Checker II, Arkray Inc., Kyoto, Japan), a convenient and accurate method in both arterial and capillary blood samples [23,24], that delivers results in a few minutes without requiring centrifugation nor transportation on ice. We collected capillary blood from the ear-lobe and placed it immediately on the reagent strip for analysis. We chose not to define an *a priori *pathological threshold for the rise in blood ammonia as there is no data on capillary blood value after such an amino acid challenge.

In a subset of 8 hospitalized patients, ammonia was determined simultaneously in capillary blood from the ear-lobe and in venous blood from a cannula inserted into the antecubital vein.

Any symptoms that appeared during or after the 60 minutes period was carefully recorded. In order to determine a physiological response to the oral glutamine load in terms of capillary blood ammonia, 13 healthy subjects (male/female: 5/8; age 54 yrs [29-72]) also underwent the test.

### Clinical outcome

All patients were followed-up for one year after the test, and all clinical episodes of HE were identified and fully characterized using patients' hospital records as well as information obtained from the family or general practitioner.

## Statistical analysis

Statistical tests were performed using Statistical Package for the Social Sciences SPSS version 10.0 (SPSS Inc. Chicago, IL, USA) Continuous variables were expressed as mean and standard deviation or median value (interquartile range [IQR]) as appropriate. The results of psychometric tests were transformed into Z-scores, which allows to normalize the value to a standard distribution curve. We used the Wilcoxon-signed rank, and chi-squared test as appropriate. To test for blood ammonia changes over time, we used the one way repeated measures ANOVA test with Dunnett's test. We constructed receiver-operating characteristics (ROC) curves to assess for overall accuracy of capillary blood ammonia levels and to identify optimal cutoffs. To identify independent predictors of development of HE episodes during follow-up, we performed a multivariate logistic regression analysis using a Cox proportional hazard model. A 2-sided p value of less than 0.05 was considered statistically significant.

## Ethical considerations

The study protocol was approved by the Ethics Committee of the Geneva University Hospitals and by the Swiss Agency for Therapeutic Products (SwissMedic) authorities. The study was conducted in accordance with the ethical principles of the Declaration of Helsinki. All patients and controls gave written, informed consent to participate.

## Results

### Prevalence of minimal HE

All patients completed the short neuropsychological tests successfully. At baseline, 25 patients (44%) met the criteria for minimal HE, while this proportion rose to 67% (38 out of 57 patients) at the evaluation performed 60 minutes post glutamine challenge (chi^2 ^: p < 0.01). Changes in psychometric tests results are given in table 2.

**Table 2:** Changes in psychometric tests after glutamine load in patients and in healthy subjects

Test patients	Baseline	60 minutes	P value
Trail test A	2.7 ± 2.3	3.4 ± 2.5	0.04
Trail test B	2.4 ± 2.1	3.5 ± 29	0.001
Pegboard	0.09 ± 1.4	0.01 ± 1.5	0.63
**Test controls**			
Trail test A	0.42 ± 1.2	0.51 ± 1.3	0.81
Trail test B	0.80 ± 1.5	0.77 ± 0.9	0.9

Note: values expressed as Z-scores.

### Oral glutamine load and blood ammonia

The oral glutamine challenge was completed in all patients and the healthy subjects. Tolerance to the test was overall excellent, although one patient presented transient nausea after 30 minutes, and one healthy subjects reported mild dizziness at 45 minutes after glutamine ingestion. There was no modification in patients' neurological status during or after the test.

**Figure 2:**
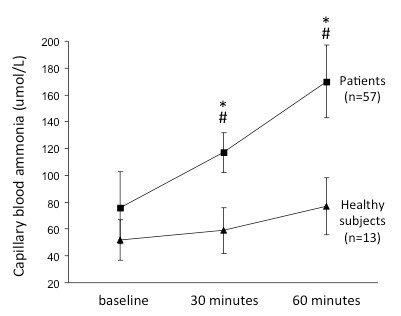
**Changes in capillary blood ammonia during the glutamine challenge in patients with cirrhosis (n = 57) and in healthy subjects (n = 13)**. # p < 0.05 versus values in healthy subjects; * p < 0.05 versus baseline values.

**Figure 3:**
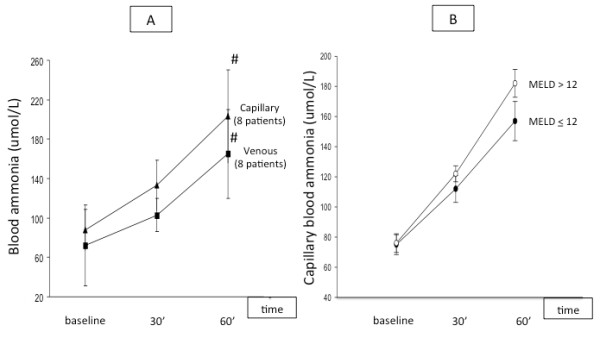
**Panel A: Evolution of ammonia in samples obtained simultaneously in capillary and venous blood in a subgroup of 8 patients with cirrhosis**. # p < 0.05 versus baseline. Panel B: Capillary blood ammonia changes over the test period in patients with cirrhosis according to the MELD score using a cut-off of 12.

We observed a progressive and significant increase in capillary blood ammonia in patients with cirrhosis, while values remained statistically unchanged in healthy subjects (see table 3 and Figure 2). In the subgroup of 8 patients who had ammonia levels determined simultaneously in two vascular beds, we observed a tendency towards higher values in capillary as compared to venous blood (see Figure 3), but without reaching the level of significance. The rise in capillary blood ammonia was not influenced by the degree of liver dysfunction using a cut-off of 12 in the MELD score (see Figure 3).

**Table 3:** Evolution of capillary blood ammonia after the oral glutamine challenge in patients and in healthy subjects

Patients	Baseline	30 minutes	60 minutes
	75.2 ± 22 umol/l	117 ± 16 umol/l*#	169 ± 51 umol/l*#
**Healthy subjects**	**Baseline**	**30 minutes**	**60 minutes**
	52 ± 11 umol/l	59 ± 15 umol/l	76 ± 23 umol/l

Note: * p < 0.05 versus baseline value; # p < 0.05 versus value in healthy subjects.

### Capillary blood ammonia and hepatic encephalopathy

**Figure 4:**
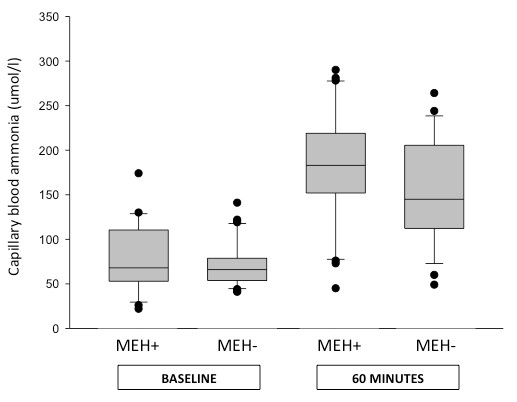
**Blood ammonia levels according to the presence (MEH+) or absence (MEH-) of minimal HE at baseline and 60 minutes aftert the oral glutamine challenge in patients with cirrhosis**.

**Figure 5:**
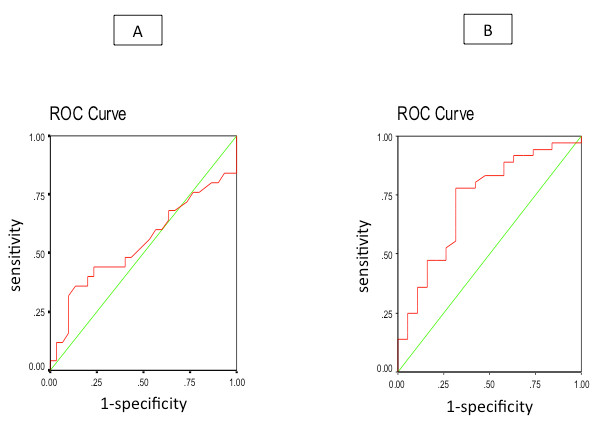
**ROC curves with respect to sensitivity and specificity of baseline (Panel A) and post glutamine load (Panel B) capillary blood ammonia levels for the diagnosis of minimal HE**. At 60 minutes, using a cut-off level of 260 umol/l, the sensitivity was 79% and the specificity was 50%.

At baseline, patients with cirrhosis, with and without minimal HE demonstrated similar capillary blood ammonia values. 60 minutes following glutamine load, there was a trend of higher values in patients with HE as compared to those without HE, but the difference was not significant (183 [45-290] versus 144 [49-264] umol/l, respectively, p = 0.133) (see Figure 4). To further explore the diagnostic accuracy of capillary blood ammonia for minimal HE, ROC curves were constructed and the area under the ROC curve (AUROC) was computed. The AUROC for making the diagnosis of minimal HE at baseline was 0.541 (95% confidence interval (CI), 0.38-0.7, p = 0.6), while at 60 minutes after the test it was 0.727 (95% CI, 0.58-0.87, p < 0.006) (see Figure 5).

The subgroup of 18 patients with previous episodes of HE had higher 60 minutes post glutamine ammonia levels compared to those who never experienced clinical episodes of HE (181 ± 13 vs 163 ± 11 umol/l, p < 0.05).

No clinically overt HE developed in the 4 patients with TIPS after the oral glutamine test. Table 4 provides the individual values of capillary blood ammonia during the test period, as well as the MHE status.

**Table 4:** Evolution of blood ammonia and MHE status in 4 patients with cirrhosis and TIPS

				Baseline		60 minutes	
Patient	Age (yrs)	MELD	Previous HE	Ammonia (umol/l)	MHE	Ammonia (umol/l)	MHE
1	66	11	+	66	+	244	+
2	57	7	-	64	-	197	-
3	48	14	+	76	+	276	+
4	71	10	-	52	-	88	-

Note: MHE+ and MHE- denotes the presence or absence of minimal HE according to psychometric tests performance.

### Incidence of hepatic encephalopathy during the follow-up

During follow-up, 18 patients, 9 of whom had a past history of overt HE, presented clinical episodes of HE at a median time of 59 days ([50-320]) after the oral glutamine test, 2 patients died of end-stage liver failure, and 3 patients underwent liver transplantation. Precipitating factors of progression to overt HE included excess of diuretics, gastrointestinal bleeding, use of psychoactive drugs and infections in 10 patients, while no precipitant could be identified in the remaining 8 patients.

The following variables were entered into a predictive statistical model for HE: age, MELD score, 60' post glutamine capillary blood ammonia, previous episodes of HE, the hepatic venous pressure gradient, and presence of esophageal varices. In the Cox multivariate regression analysis, only the MELD score (HR 1.122; 95% CI, 1.018-1.236) and previous episodes of HE (HR 3.2; 95% CI, 1.069-9.58), but not capillary blood ammonia, were independent predictor of development of overt HE during follow-up. Using baseline or 30' post test ammonia levels in the model did not modify the results of the analysis.

## Discussion

Making a diagnosis of minimal HE is challenging for the clinician, who needs a sensitive, reliable, and easy-to-use diagnostic tool. However, at present, neuropsychological evaluation and electrophysiological tests do not fulfill these requirements. This aspect may explain in part why only a minority of hepatologists are screening for minimal HE in daily practice [25]. Therefore, a simple test would be welcome and greatly facilitate the diagnosis and thus the management of HE. In our study, we tried to improve the diagnostic accuracy of blood ammonia, a key substance in the pathogenesis of HE, for the presence of minimal HE. To do so, we used capillary blood as an equivalent to the arterial compartment, and induced hyperammonemia *via *small intestine ammonia production to unmask HE. Finally, we designed the test as a bedside procedure fulfilling the definition of POCT to improve patients' and clinician's acceptance.

First of all, using our short test battery, we confirmed the high prevalence of minimal HE (44%) in our patients with advanced cirrhosis (82% of Child B and C), consistent with published data [3,4]. Secondly, we demonstrated that an amino acid load using oral glutamine at a higher dose than used in previous studies [26,19] is well tolerated in patients with moderate to severe liver insufficiency. We also observed that the present test didn't precipitate any clinical episodes of HE in the 4 patients with TIPS, as this situation worsens portal-systemic shunting and makes the patient more vulnerable to the HE [27]. Our results also demonstrate that capillary blood ammonia is not an accurate biological marker of minimal HE in patients with cirrhosis, nor a reliable predictor of future episodes of overt HE during follow-up. Nevertheless, we show that the diagnostic value of capillary blood ammonia can be substantially improved following an oral aminoacid load, raising the AUROC value from 0.541 at baseline to 0.727 at 60 minutes after the oral glutamine challenge. This significant and rapid elevation in blood ammonia after the test is consistent with the significant contribution of the small intestine to the production of ammonia [28], in addition to the well-accepted role of colonic bacteria. The presence and severity of portosystemic venous collaterals is another important parameter that determines blood ammonia levels [7]. Ortiz et al. [29] reported a similar increase in capillary blood ammonia following an oral glutamine challenge in patients with congenital portosystemic shunts as compared to patients with cirrhosis. In our study, most patients had evidence of portosystemic collateralization visible on different imaging modalities preventing an accurate analysis, therefore we did not include this parameter nor the value of portal pressure in the statistical model.

Induced hyperammonemia using either glutamine alone [22] or combined with other amino acids [30] is associated with electroencephalographic, brain magnetic resonance and biochemical alterations, the neuropsychological consequences of which are inconstant, showing either absent [31], partial [22] or generalized [32] deterioration of tests' performance. In the present study, we observed changes limited to the Trail A and B tests, both commonly used components of standard test battery for the detection of minimal HE [3], suggesting that visuo-spatial skills and visuo-motor coordination are affected by hyperammonemia. We also explored whether induced hyperammonemia could predict the development of overt HE during follow-up, and found that post test capillary blood ammonia was a poor predictor of events. This is in contradiction with results from the study by Romero-Gomez et al. [19] who reported that an abnormal ammonia response after oral glutamine was predictive of the development of overt HE during follow-up. The distribution of liver disease severity in the 2 studies (Child A patients: 75% versus 17% in our study) and the absence of any treatment for HE that may influence the ammonia response following a glutamine challenge [33,34], could explain these differences.

Our study is in line with previous results which showed that determination of blood ammonia levels, either a single venous dosage [18] or in capillary blood after induced hyperammonemia, correlates only poorly with symptoms of HE. Whether determination of ammonia in a arterialized-venous blood, as in our study, shows higher values as compared to venous blood has been proposed [16] but not confirmed in our small subgroup of 8 patients. Thus, additional parameters such as size and extension of portosystemic shunts [29,15], hyponatremia [35], nutritional status [36], the presence of hepatitis C infection [37], together with the presence of a systemic inflammatory reaction syndrome (SIRS) [38] that may modulate ammonia neurotoxicity at the cerebral level, are factors that influence the development of HE. We were however not able to examine all these parameters in our cohort, as values of serum creatinine, sodium, and C-reactive protein were available only in a limited number of patients.

## Conclusions

In conclusion, we demonstrated that determination of capillary blood ammonia after a bedside oral glutamine challenge is feasible, well tolerated and superior to basal levels for the diagnosis of minimal HE in patients with cirrhosis and moderate to severe liver failure. The increase in blood ammonia, absent in healthy subjects, correlated imperfectly with the deterioration in psychometric tests. Thus, in spite of a recognized important pathogenic role in HE, blood ammonia, even in a capillary, arterialized vascular bed, remains an approximate marker for the presence of HE. According to our data in unselected patients with cirrhosis, ammonia determination in capillary blood after an oral glutamine challenge is not a valid tool for the diagnosis of HE and a poor predictor of future clinical episodes of HE.

## List of abbreviations

HE: hepatic encephalopathy; MELD: model for endstage liver disease; TIPS: transjugular intrahepatic shunt; HVPG: hepatic venous pressure gradient; POCT: point-of-care testing; AUC: area under the curve.

## Competing interests

The authors declare that they do have nothing to disclose regarding funding or conflict of interest with respect to this manuscript.

## Authors' contributions

SD: participated in the development of the study, inclusion and follow-up of patients, performance of psychometric tests and blood ammonia dosage, and drafting of the manuscript. EG: participated in the selection of patients and drafting of the manuscript, and performed the statistical analysis. PRB: participated in the development of the study, supervised the psychometric tests, and drafted the manuscript. NG: participated in the development of the study and drafting of the manuscript. GM: participated in the selection and inclusion of patients. AH: participated in the development of the study, selection of patients and drafted the manuscript. LS: designed the study, included patients, performed psychometric tests and ammonia dosage, and drafted the manuscript.

All authors read and approved the final version of the manuscript.

## Pre-publication history

The pre-publication history for this paper can be accessed here:

http://www.biomedcentral.com/1471-230X/11/134/prepub
